# One tool to find them all: a case of data integration and querying in a distributed LIMS platform

**DOI:** 10.1093/database/baz004

**Published:** 2019-01-30

**Authors:** Alberto Grand, Emanuele Geda, Andrea Mignone, Andrea Bertotti, Alessandro Fiori

**Affiliations:** 1 Department of Oncology, University of Torino; 2 Candiolo Cancer Institute - FPO IRCCS, Strada Provinciale, Candiolo, Italy

## Abstract

In the last years, Laboratory Information Management Systems (LIMS) have been growing from mere inventory systems into increasingly comprehensive software platforms, spanning functionalities as diverse as data search, annotation and analysis. Our institution started in 2011 a LIMS project named the Laboratory Assistant Suite with the purpose of assisting researchers throughout all of their laboratory activities, providing graphical tools to support decision-making tasks and building complex analyses on integrated data. The modular architecture of the system exploits multiple databases with different technologies. To provide an efficient and easy tool for retrieving information of interest, we developed the Multi-Dimensional Data Manager (MDDM). By means of intuitive interfaces, scientists can execute complex queries without any knowledge of query languages or database structures, and easily integrate heterogeneous data stored in multiple databases. Together with the other software modules making up the platform, the MDDM has helped improve the overall quality of the data, substantially reduced the time spent with manual data entry and retrieval and ultimately broadened the spectrum of interconnections among the data, offering novel perspectives to the biomedical analysts.

## Introduction

The introduction of automation and high-throughput technologies in laboratory environments has raised diverse issues related to the amount and heterogeneity of the data produced, the adoption of robust procedures for sample tracking and the management of computer-based workflows needed to process and analyze the raw data. Laboratory Information Management Systems (LIMS) have gained increasing popularity because they can ensure good levels of quality control over the laboratory activities and efficiently handle the large amounts of data produced (1).

LIMS aim at assisting the researchers in their daily laboratory practice, improving the accessibility of the instruments and tracking biological samples and their related information.

In the past decade, several open-source as well as proprietary LIMSs have been developed. Commercial solutions are typically large, complex and feature-rich products designed to easily support large laboratories. Their license fees can be prohibitive, and extra features may come at additional costs (2). To reduce these costs, the last generation of commercial LIMS adopt web-oriented software technologies, particularly the software as a service distribution model, which reduces the customer’s final expenditure on license fees, hardware and maintenance. Examples of commercial solutions are STARLIMS (3), Exemplar LIMS (4) and LabVantage SAPPHIRE (5).

Commercial LIMS tend to offer features based on common laboratory procedures and best practices, which may not fit highly specific settings well. For instance, LabVantage SAPPHIRE provides a large set of features, such as sample and batch management, Quality Control, advanced storage and logistics and task scheduling. However, the life cycle of xenopatients (i.e. biological models for cancer research based on the transplantation of human tumors in mice) is not available in the standard software and should be implemented as a custom module by the software house. Another issue that affects commercial LIMSs is the management and standardization of genomic data. To the best of our knowledge, these systems do not exploit any knowledge base related to the genomic data and do not provide any validation and analysis of different genomic data stored in the system.

Other products focus instead on specific sub-domains, such as Galaxy LIMS (6, 7), addressing DNA sequencing and annotation, or SeqWare (8), tracking *in vivo* and *in vitro* experiments and building complex analysis workflows.

For this reason, many institutions have invested in the development of in-house solutions and/or have adapted open-source projects to their own requirements. In this way, the developed solutions can provide functionalities that meet the specific needs of the researchers in their institution laboratories. From an engineering perspective, developing in-house solutions may also permit to explore and adopt new technologies, in order to define better data models and improve system performance.

To address a substantial mismatch between the LIMS solutions on offer and the functional requirements dictated by research practice, in 2011 the Institute for Cancer Research at Candiolo (Italy) started to implement its own LIMS, named the Laboratory Assistant Suite (LAS) platform (9). The main purpose of the platform was to assist researchers in different laboratory and research activities, allowing management of different kinds of raw data (e.g. biological, molecular), tracking experimental data, supporting decision-making tasks and integrating heterogeneous data for complex analyses. As development progressed, several new features and modules were included to (i) track clinical data, (ii) include support to the newest technologies exploited for molecular experiments and (iii) standardize the description of genomic data by means of semantic web technologies. Thanks to these new features, scientists can gain better insight into tumor development by jointly studying the clinical evolution of the disease and the experimental results derived from *in vivo*/*in vitro* experimentation. The experimental pipelines exploited in the translational research context are the primary focus of the LAS, which targets the standardization of the genomic data to allow a comparison of results coming from different technologies.

Still, unlike the other commercial and open-source platforms, the LAS makes an attempt at covering a wide range of diverse laboratory procedures and, thanks to its versatile and general-purpose structure, it can be extended to support new ones with a limited effort.

Thanks to the vast variety of different experimental technologies supported by the LAS and their high level of specificity, large amounts of heterogeneous and complex information are collected in separate databases. To enable the users to extract and correlate information from the different databases exploited by the platform, a Multi-Dimensional Data Manager (MDMM) module was developed. The module takes care of merging data from the different LAS databases, and provides a simple graphical user interface to extract the information of interest without any knowledge of a query language. A tool to visualize biological entities and their related information with a hierarchical tree structure is also available, while other powerful visualization tools are currently under development. To the best of our knowledge, no similar tools applied to biological data and distributed databases exist.

The paper presents the main characteristics of the LAS and its exploitation in the research laboratories of the Institute for Cancer Research at Candiolo and its research partners. Next, the main features of the MDDM are described. Finally, current and future research directions are presented.

## LAS

Translational research aims at enhancing patient care, transferring scientific discoveries from the laboratory to a real clinical context. It is a kind of metaphorical scientific cycle from bench to bedside and back again through complex iterative processes, operating between laboratory (i.e. preclinical research) and clinic. To the aim of managing and integrating preclinical and clinical information, a robust but flexible data management platform is needed. In particular, different types of information (e.g. biological data, molecular data, procedure tracking data and sample tracking data), some of which can be highly complex, should be independently managed by the platform but, at the same time, interconnected to permit integrated analyses.

The LAS platform is freely available upon request to the authors. The software is distributed by means of a Docker-based approach to allow interested organizations to configure it according to their constraints. Moreover, the exploitation of Docker allows system administrators to run the software on different servers using the Docker Swarm configuration for balancing the workload as well as data resources. We usually recommend to install the LAS on at least two servers, one dedicated to the containers running the software and the other for databases. The servers characteristics depend on several aspects, such as the number of simultaneously logged users, the number of biological entities tracked and the dimension of raw data stored. We suggest as initial setup a server with at least 16 GB of RAM and a storage space of 2 TB. Interested users may refer to the video tutorials (available at the URL: http://lasrep.ircc.it/) to explore main system features and as a reference guide during its usage. The documentation of the platform is provided with the software and can be downloaded from the documentation section of the LAS instance of the Institute for Cancer Research at Candiolo (https://las.ircc.it/).

In the following, we present the data architecture and the main functionalities included in the platform.

### Data models

The LAS platform has been developed using different database technologies to fit the needs of the application, and to handle in a suitable way the heterogeneous data characteristics.

The platform makes use of relational databases to track biological entities and their properties, and the information about the various experimental procedures. Since the platform includes different modules managing substantially different types of entities and/or specific laboratory procedures, different database instances are exploited. The core biological entities (i.e. Aliquot, Biomouse and Cell Line) are identified by a unique and mnemonic key, named GenealogyID, that encodes relevant information regarding the history of the entity. This key is automatically generated by the LAS platform through formal rules and may be used to link the data across the databases.

Parallel to the relational databases storing operating data, a graph database is exploited. It is used to represent the complex inherent hierarchy of biological entities and their relationships. Being able to easily and efficiently reconstruct the genealogical tree of each entity is indeed an essential feature of the platform, allowing the user to perform *ad hoc* queries and to isolate specific sub-trees of biological entities involved in the experimental pipeline. Moreover, the graph database has been exploited to store a knowledge base for the heterogeneous domains managed by the LAS modules. By using a graph representation, all these domains can be easily interconnected, while the knowledge base can be continuously updated and augmented with new layers of information and different levels of abstraction (e.g. proteomics, clinical, etc.). Finally, a social network of users and research groups using the LAS platform is also stored in the graph, to model data ownership, resolve data access conflicts and manage data sharing and collaboration among different groups or users.

A document database, MongoDB, is also used to store files associated with biological entities and metadata generated by both the LAS Genomic Data Manager modules and the MDDM. The latter usage will be discussed in detail in the next section.

### Functionalities

The LAS architecture includes a set of software modules, i.e. fully-fledged web applications, each addressing a different type of biological entity and its associated experimental procedures. Modules may interact with each other by means of web APIs, e.g. to exchange data and/or to carry out operations that span multiple entities or domains. The modules currently included in the LAS platform are described in the following.

Even if the platform has been developed since 2011, we always took into account security issues during the design and development processes of the platform. In this way, our software is compliant with the constraints of the General Data Protection Regulation (GDPR), enforced on 25 May 2018. Indeed, the management of data produced by different users and/or groups requires that the access to functionalities and information are restricted according to several criteria such as group and/or project membership, and user role. For these reasons, the platform manages users and their privileges following these concepts:

- Working Group: A Working Group (WG) is a set of users in the LAS platform that work together toward a specific goal (e.g. project, research activity). The data produced by the users of the same group are private, unless they intentionally share data with other groups.
- User Profiles: Each user belonging to a WG has a set of permissions to access the LAS functionalities they have been enabled to use. These functionalities are defined according to the role selected during the user registration process. The manager of her/his WG or the system administrator can assign new functionalities upon request.

To collect data, the user is supposed to specify the Informed Consent signed by the patient for specific research activities (e.g. preclinical trials) involving personal samples and information. This document is defined by a committee to accomplish all the constraints included in the GDPR. Since the data are collected for research purposes, the patient can only revoke the usage of the biological samples, but not the information (e.g. experimental results) collected by the researchers. Only the researchers that are included in the research project can manage these samples and track the experimental processes according to their profile. The platform tracks all the procedures performed by each user in order to identify malicious usage of the software.

The Clinical Module tracks for each patient both context information (i.e. personal data, Medical Center of the Trial, etc.) and relevant clinical events through Case-Report-Form. All data are related to the relative Informed Consent that grants data and specimens sampling.

The BioBanking Management Module covers a wide range of activities, including management of biological samples and associated pathological information, as well as support to a number of laboratory-related procedures. For instance, the module can handle the collection of biological material from surgical intervention and the acquisition of aliquots from external laboratories. Aliquots stored in the system are characterized by features such as tumor type (e.g. colorectal), tissue type (e.g. liver metastasis), source hospital or laboratory and pathological information. Measurement of aliquot physical characteristics, such as volume, concentration, purity and quality can be tracked by the module, as well as the derivation of new biological materials (e.g. DNA and cDNA) and the planning of molecular experiments.

The biological material used in our laboratories is stored by means of several types of containers (e.g. freezers, racks, plates and tubes). Their mutual interactions (i.e. which types of containers can host other containers) can change according to characteristics such as the layout and the laboratory procedure. The Storage Management Module allows managing any kind of container by defining and applying different rules to them, and tracks the relationships between the containers and the biological entities.

Different types of molecular analyses can be conducted on biological samples, to investigate various aspects of their genetic constituents that may have an impact on the development of oncogenic behavior. For instance, biologists may be interested in analyzing mutations for a target gene involved in tumor proliferation. In an effort to closely track the translational research pipeline from the collection of samples to their analysis, the LAS provides support to tracking the most frequently used molecular profiling techniques in our institution [e.g. Sanger sequencing, real-time polymerase chain reaction (PCR) and Sequenom]. Each molecular module queries the Knowledge Base of the Genomic Annotation Manager (GAM) to retrieve the description of its reagents, as well as a specification of all possible alterations (e.g. sequence alterations and gene copy number variations) known in the literature, to allow both the experiment definition and the evaluation of experimental results.

The GAM provides a higher-level, qualitative insight into the genomic features of biological samples. This information is shaped in the form of annotations, i.e. a set of semantic labels attached to a sample, pointing out some of its relevant features. To ensure semantic coherence and adopt a standardized nomenclature, all relevant concepts from the genomic and biological domains used for labeling samples have been drawn from a number of public, freely accessible databases and ontologies (10–13). This information has been structured into a knowledge base, modeled as a graph and stored in a graph database (14). Concepts are interlinked with one another, according to both general-purpose semantic relationships such as containment (`part of’) or generalization (`is a’), and domain-specific relationships (e.g. indicating an underlying biochemical process, as in `is transcribed from’). New concepts and relationships, as well as new domains of interest, may be added or layered as needed, to account for novel findings and broaden the spectrum of investigation. Within the GAM, every annotation is a semantic statement establishing a relationship, expressed by means of a predicate, between a biological sample (the subject of the statement) and a concept (the object of the statement), such as a genetic mutation. It is represented within the graph database as a node of type `annotation’ with a pair of incoming and outgoing edges—one linking the biological sample to the annotation node by means of a `has_annotation’ relationship, and the other linking the annotation node to the reference node in the knowledge base by means of a `has_reference’ relationship. The annotation node is often linked to other nodes, such as the process that produced the annotation or the raw experimental data.

Finally, the platform allows tracking *in vivo* and *in vitro* experiments. `*In vivo*’ (Latin for `within the living’) studies are those in which the effects of various biological entities are tested on whole, living organisms—usually animals (e.g. xenopatients). This kind of experiments are normally exploited to test drug therapies and expand the collection of biological samples. We based our development on the model described in (15) to manage immunocompromised animals and monitor the xenopatient life cycle, from their acquisition by the research institute to their death. Unlike *in vivo* experiments, *in vitro* studies are performed with cells or biological molecules studied outside their normal biological context. The LAS platform defines a Cell Line as the set of biological entities that are generated from the same biological entity and are under the same experimental conditions. The experimental conditions are defined by the protocols that describe the type of process (i.e. adherent, suspend and organoid) and the set of culturing conditions applied (e.g. nutrients and chemicals, hormones/growth factors and antibiotics). In addition, the platform allows the management of the generation and thawing procedures of cell lines.

**Figure 1:**
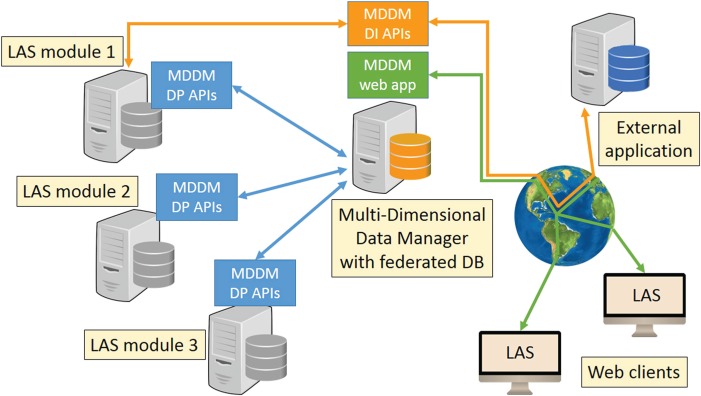
MDMM architecture.

## MDDM

Besides tracking experimental procedures and recording all the data related to biological entities, the retrieval of the information tracked by the platform is fundamental to discover new knowledge related to tumors. Due to the large number of laboratory activities and biological entities managed by the LAS with a high level of specificity, and to streamline the development and maintenance process over time, each LAS module operates on a separate database instance, storing both operational data and domain-specific knowledge, and partially replicating key information from other LAS modules. As a result, while each instance focuses on its own specific subset of functionalities, no one database can provide a comprehensive insight into the data. Nonetheless, building actionable knowledge requires the integration of heterogeneous information to establish connections among different and related biological entities, or to explore different facets of the same entities, both for operational and for research purposes. For instance, identifying the number of tubes stored in each plate of a given freezer and their content may be required for managing physical storage equipment. From a researcher’s perspective, the study of the evolution of the tumor mass in a mouse under pharmacological treatment (monitored by means of *in vivo* experimentation and tracked by the Xenograft Management Module) must be traced back to the originating patient (managed by the Clinical Module), and should be further correlated to the mouse genomic mutational status and gene expression levels (collected by the set of Molecular Modules and annotated by the Genomic Annotation Module). The MDDM addresses the issue of extracting all information of interest stored in the databases of each LAS module, providing the end user with an intuitive graphical tool for building customized queries with a unified view on the entire collection of databases. In addition, the MDDM can be exploited to run programmatical queries via a set of web APIs, enabling other LAS modules, as well as other external applications, to retrieve data of interest.

### Architecture

The MDDM has a distributed architecture. Its main components, shown in Figure 1, are the central MDDM Data Integrator (DI) module and the distributed MDDM Data Provider (DP) modules. The DI module provides a unified logical view on the databases of the other modules, thus acting as a (read-only) federated database (16). The DP modules rely on a standalone application layer that must be installed within each LAS module, allowing the LAS modules to join the federated database. Interactions between the DI and the DP modules occur via two distinct sets of APIs.

The MDDM DP APIs are called by the central DI module. They provide an interface to perform the following tasks. (i) Retrieve schema information from each LAS module’s database. This operation is executed once at setup time, when the LAS module is initially registered as a member of the federated database. The APIs collect schema information (i.e. database entities, entity attributes and relationships among entities) from the object-relational mapping layer provided by Django, the Python-based Model-View-Controller (MVC) development platform of choice. Thus, they are independent of the choice of the actual DataBase Management System (DBMS) adopted as a storage backend. These metadata, stored by the DI module in its local database, will later be used to define the logical schema of the federated database (see Section Logical design). (ii) Run SQL queries on the LAS modules’ local databases and return data to the DI module. Query requests are sent by the DI module using an *ad hoc*, SQL-injection-proof protocol. To ensure higher flexibility and allow more complex operations not well supported by the ORM layer, the API speaks directly to the underlying DBMS using raw SQL queries and a DBMS-specific library handling the slight syntax variations among products. Currently only a library for the MySQL database management system is available. See Section Query execution for further details about the query engine.

The MDDM DI APIs provide a programmatic interface for executing complex queries on the federated database. The queries that may be executed through this interface are currently not free-form. Instead, they are template queries that receive zero or more parameters and return a set of rows by implementing a user-defined query flow. These template query flows may be designed by the LAS users by resorting to the Query Generator Graphical User Interface (GUI) (see Section Query Generator for details). Thus, an unlimited number of different queries may be designed and run through this interface. In addition to an endpoint for submitting query requests, the DI includes a set of APIs for obtaining the list of available templates, and a structured description of their input parameters and output schema. In this way, hard coding of result parsing routines can be avoided or at least reduced, since the data consumer can automatically detect changes in the input parameters or in the output fields, and take appropriate measures. The DI APIs serve two main scenarios. (i) They are used by LAS modules that need additional information from other modules to accomplish their tasks. The DI provides them with a unified, possibly aggregated set of information by running an appropriate query. (ii) They may be used by external services or applications that need to query the data stored in the LAS platform (e.g. collaborations with research partners, running external pipelines or scripts).

### Logical design

The MDDM provides a logical abstraction of the databases of the different LAS modules taking part in the federated database, which may be accessed seamlessly as a single database. Initially, the MDDM DP APIs are installed in each relevant LAS module. Next, each module’s database is registered in the MDDM as a DP through an *ad hoc* GUI, requiring the URL of the LAS module (which may reside on a different server) and a number of configuration parameters (such as a name, colors to be used by the Query Generator GUI to draw the corresponding entity buttons, etc.). The DP’s schema is contextually imported through the DP API. Before the DP may be queried, a number of Entities and Query Paths must be defined by an experienced user (usually the database administrator, or an experienced PI) using a dedicated administrative GUI. Entities are classes of concepts and/or objects that are relevant in the given context, similar to their namesakes in the Entity–Relationship logical database model (17). Within the MDDM, each Entity is based on an existing table from the original DP database, but in order to enhance its expressiveness, its attributes are not limited to those included in the original DP table. Instead, any attribute from a related (under the constraint that the base table has a one-to-one or many-to-one relationship with the related table) DP table may be used or combined with other attributes to form an Entity attribute. Entity attributes can be used to define filtering parameters and/or output attributes for the Entity. Query Paths generalize the relational notion of a foreign key, in that they dictate how two different Entities A and B should be related to each other. They are defined as the set of DP tables that must be joined, through their foreign keys, in order to link the base table of Entity A to the base table of Entity B. Given an instance of Entity A, the maximum number of instances of Entity B that correspond to it can be equal to one (if the foreign key chain only includes many-to-one or one-to-one relationships) or larger than one (if it includes at least one one-to-many relationship). Query Paths can be defined through an *ad hoc* interface. To guide the user throughout this task, the MDDM can automatically build a graph, whose nodes are DP tables and edges are relationships between tables, and exploit a shortest path algorithm to identify an optimal Query Path between a pair of Entities. Alternatively, Query Paths can be manually built by selecting the available foreign key relationships.

**Figure 2:**
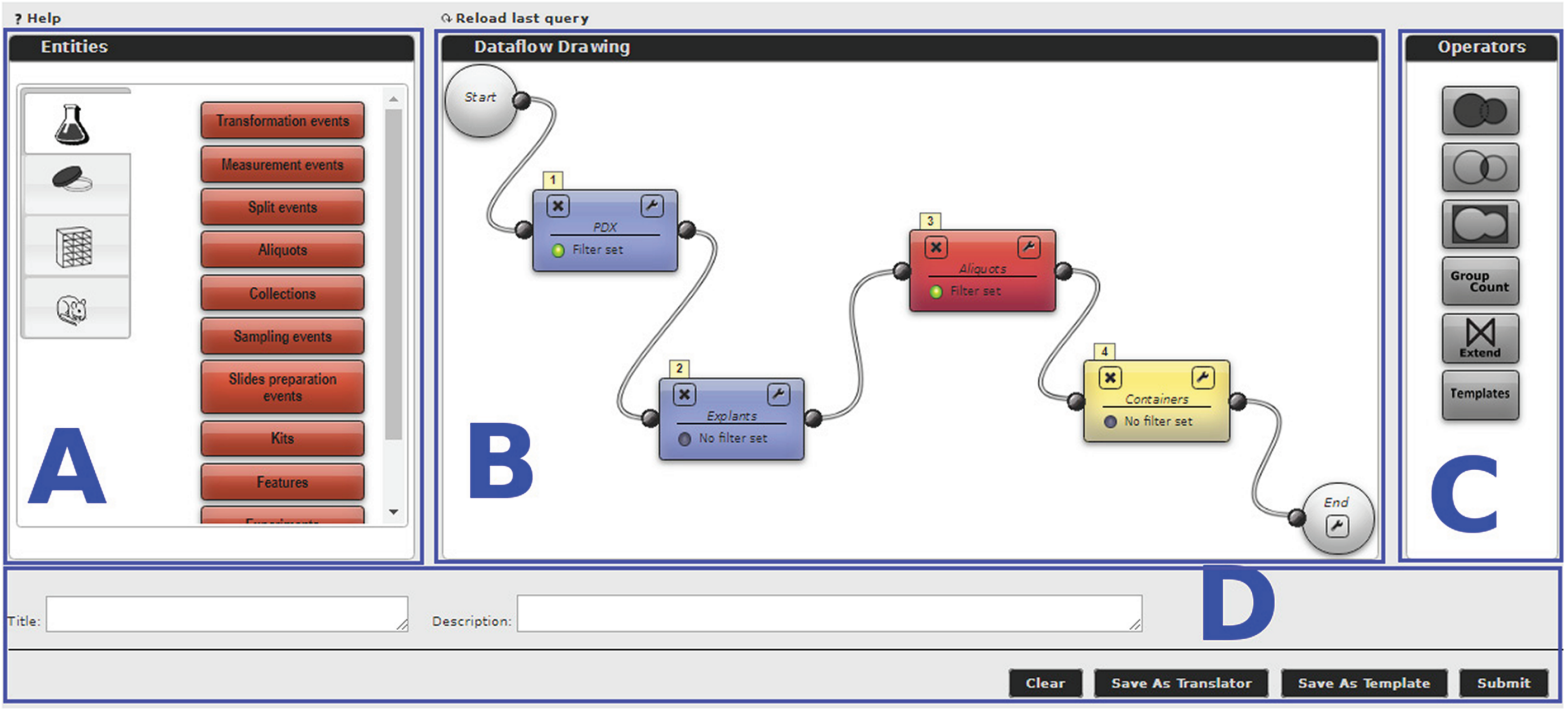
Query Generator interface.

### Query Generator

The MDDM’s core feature is a tool to design queries. The tool exploits a graphical metaphor based on cascaded blocks that should be easily understood even by inexperienced users. Each query block represents one of the federated database Entities, as previously described, or an operator, and it defines which kind of object will be returned as the block’s output. Available query blocks are shown on the left-hand side of the editor (block A in Figure 2) and categorized according to the DP from which the data are drawn (e.g. the flask icon for Biobank data and the mouse icon for xenopatient data). The user can drag and drop the query blocks on the workflow editor (block B). For instance, Figure 2 shows the xenopatients, also known as patient-derived xenograft (PDX) models, and Explants blocks from the Xenopatient DP, the Aliquots block drawn from the Biobank DP and the Containers block from the Storage DP. The reported query aims at retrieving all the containers (e.g. tubes) carrying aliquots explanted from PDX models. Filtering conditions may be specified for each block by clicking the funnel icon. In the reported example, the user can retrieve only the viable aliquots that are still available. Set operators (Union, Intersection and Difference) and special operators (Group-count, Extend and Template blocks), listed on the right-hand side (block C in the figure), can also be used. Blocks must be interconnected to build a query workflow by drawing a wire between a block’s output terminal and another block’s input terminal. Two blocks may only be connected if a Query Path exists (as defined by the MDDM administrator) that instructs the system how to match the corresponding Entities. Conversely, an error message will be shown. Once a workflow has been defined, the user may assign a title and a description to it (block D), then either run the query immediately, or save it in the system as a template, so that it can be run later either through the Query Generator Template block, or through the DI APIs. Furthermore, queries may be saved as Translators, i.e. a special type of template that may be optionally run for every row appearing in the result set of a query, to enrich it with additional, related information. Query results are displayed in a paged tabular structure. Since they are stored in the document database (MongoDB), they can be reopened at any time without re-running the query. In addition, they can be exported in multiple formats and saved locally by the user.

By using the Query Generator, the users can build any kind of query disregarding the structure of the databases, the query language and the low-level procedure to connect data among several databases. For instance, they can retrieve information about treatments performed on xenopatients starting form a set of aliquots, the location of aliquots generated by cell lines, the vital status of mice belonging to a specific project and many other data valuable for daily work and for statistical analyses.

### Query execution

The query flow designed by means of the Query Generator is structured as a sequence of interconnected blocks. Internally, the query is represented as a tree, whose root node is the end terminal and whose leaves are the start terminals of each initial block. Thus, queries are executed by depth-first traversing the query tree in post-order. For each visited query block, the MDDM federated database is accessed to identify the underlying DP tables. The query block is translated into a relational query structure by instantiating the required DP tables, setting the appropriate join conditions, applying the query block parameters as filtering conditions and adding aggregation operations (if any). The choice of attributes selected from the DP tables as the block outputs depends on whether the query block is an intermediate block (thus only requiring the primary key and any correlation attributes) or the final block (requiring all attributes defined as outputs for the block). In addition, the Query Path linking the current block to the next one is loaded, and any other required DP tables are also instantiated. Next, the query is sent to the DP through the APIs. If both the current block and its successor reside in the same DP, the DP APIs will not issue a real query to the underlying DBMS, but will create a logical view wrapping the query. Conversely, if the successor block belongs to a different DP, a query is issued, and result rows are returned to the MDDM. When backtracking to the successor block, the results are sent to the DP together with the new query and inserted in an indexed temporary table that is joined with the rest of the query schema. This generally improves the performance of cross-DP queries. In many cases, the Genealogy ID is used as a key to link Entities belonging to different DPs. As a last step, translator templates selected by the user (if any) are run on the result set. Template queries are managed by storing the tree structure of the corresponding query in the MDDM database. The tree is reloaded at each template execution, and its parameters are populated. Next, the tree is traversed as previously described.

**Figure 3:**
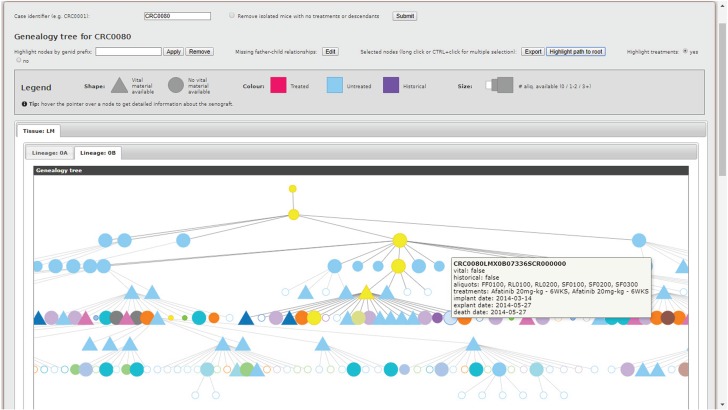
Genealogy Tree Visualizer interface.

### Visualization tools

As outlined in the introduction, translational pipelines, i.e. the laboratory practice of transplanting tumor specimens into different organisms or environments to study their proliferation, are of vital importance to cancer research. Therefore, practitioners need effective tools to examine the complex hierarchies of biological entities involved in the pipelines. The MDDM addresses this requirement with the Genealogy Tree Visualizer. The rationale is to leverage the intrinsic hierarchical structure of the xenopatient pipeline to produce a graphical tree representation capable of highlighting (i) father–child relationships among xenopatients, and (ii) multiple layers of relevant features for each individual, by exploiting a succinct but intuitive visual encoding relying on shapes, sizes, colors and tooltips. When the user inputs a collection identifier using a Genealogy ID prefix (e.g. CRC0080), the system extracts relevant data from the MDDM federated database by exploiting dedicated templates, run through the DI APIs. Thus, by modifying such templates, the tool can be easily extended to integrate and visualize additional information. Next, xenopatients are automatically partitioned based on different features (i.e. the type of tissue from which the original human tumor was collected, and the lineage), and individual hierarchical trees are constructed. The result is directly shown to the user, who may customize the visualization to her liking.

An example is shown in Figure 3. Some of the features of each xenopatient are always visible (i.e. the shape, indicating the availability of vital material for the xenopatient, and the size, indicating the number of specimens collected from the xenopatient and available in the biobank), while others, exploiting different color coding schemas, can be layered on demand. The tool can currently visualize or highlight (i) treated, untreated and historical (i.e. prior to the introduction of the LAS platform and, hence, not comprehensively tracked) xenopatients, (ii) experimental treatment protocols applied by practitioners, (iii) xenopatients satisfying certain treatment and biological material availability conditions and (iv) the path from a given xenopatient to its root ancestor. In addition, statistics about treatment protocols are provided (not shown in Figure 3), whereas details about each xenopatient is displayed as a tooltip when hovering over the corresponding node. To limit the number of individuals, which can become overwhelming, the tree may be pruned by removing disconnected nodes without any treatments. Furthermore, father–child relationships that are missing for historical xenopatients can be manually defined.

**Table 1:** Aliquots statistics based on their type

	**Human**	**Xenopatients**	**Cell lines**
**Complementary DNA**	0	1414	74
**Complementary RNA**	14	1470	0
**DNA**	17 966	28 240	945
**Formalin fixed**	1454	22 911	336
**Frozen**	733	0	0
**Frozen sediment**	78	0	0
**Labeled section**	106	3192	0
**OCT frozen**	1	338	0
**Protein**	0	0	129
**Plasma**	36 220	185	0
**Paraffin section**	107	3478	0
**RNA Later**	9455	56 493	11
**RNA**	379	8449	617
**Snap frozen**	1453	49 282	134
**Viable**	8901	30 126	10 032
**Total**	**76 867**	**205 578**	**12 278**

**Table 2:** Number of aliquots used for each experiment

**Experiment**	**#Aliquots**
Beaming	33
DigitalPCR	33
Histology-IHC	3939
MicroArray	276
RealTimePCR	3764
SangerSequencing	808
Sequenom	10 508
WesternBlots	75
Collaboration (external)	11 264

## Platform usage

**Figure 4:**
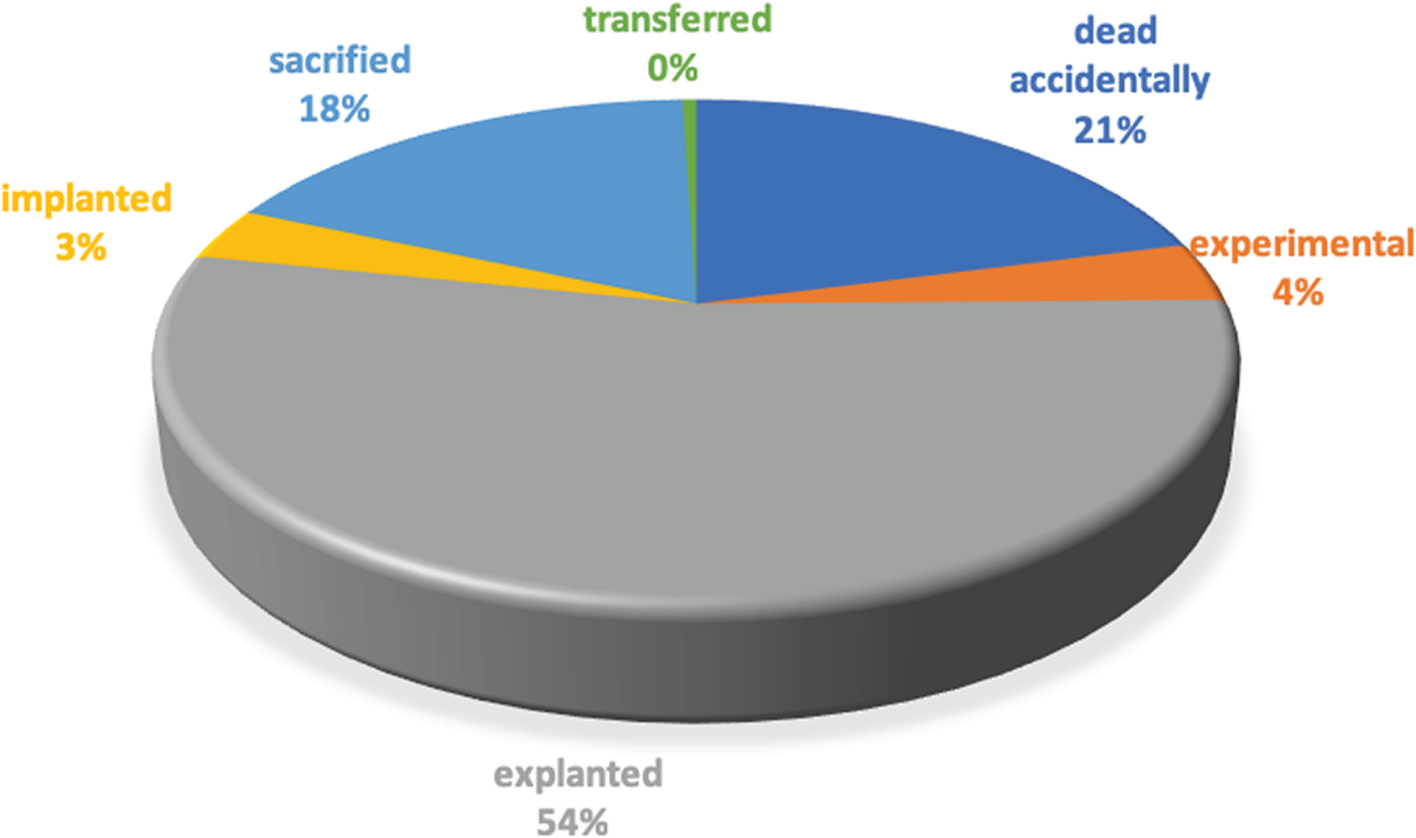
Mice status.

The LAS platform has been actively employed for research in the Institute for Cancer Research at Candiolo since March 2012. Only a few modules (e.g. BioBanking, Storage) have been available in the first release, with a subset of the current features, while the modules addressing the molecular experiments were introduced in 2013. During the last years, more functionalities have been developed to improve the user experience and provide new functionalities to track more information about biological entities and their life cycles. Within our institution, a number of research groups are currently using the platform, and a large amount of data has already been produced and stored. In particular, as of November 2018, the researchers defined approximately 10 800 collections, each one including all biological entities (i.e. aliquots and PDXs) that share a common origin (i.e. the same collection event). The BioBanking module currently stores approximately 300 000 aliquots of different types, as pointed out in Table 1. On average, 3200 aliquots are generated each month from surgical interventions on human patients, xenopatients, cell line thawing and derivation procedures. Since the most active user group works with PDXs, ~70% of the aliquots stored are generated from mice. Derived aliquots represent ~20% of the BioBanking content, and most of them are DNA. The platform also tracks aliquot consumption according to the types of experiments as reported in Table 2. Approximately 37% are sent to external laboratories to perform special analyses, while all the other molecular experiments are performed in our institution. The *in vivo* experiments represent one of the main activities of our institution as well as the plasma collection. Indeed, more than 44 000 mice have been tracked. Most of them have already been explanted to generate new aliquots, while approximately 1700 mice are currently under treatment with experimental drugs and 1500 are now implanted to expand the biological material. A detailed categorization of mice statuses is reported in Figure 4. At the moment of writing, more than 15 000 experimental treatments have been applied and 143 are still under execution.

The MDDM module has been widely used in the past years. We tracked more than 15 000 queries submitted by the users from the Query Generator. This highlights the relevance of this LAS module in the daily activities of our institution.

The LAS platform has been changing the research activities in our institution. We periodically ask users about their opinion on diverse system features to understand their impact on the daily work of researchers. Usually, they answer that the system is proving very useful in supporting their research, even if they met some difficulties when they started using the platform. Indeed, they admit that the LAS has changed their working habits (e.g. users of the platform must follow predefined rules and procedures for each operation, and instantly report their activity). Furthermore, they noticed an improvement in the data quality and a reduction of the time spent in tracking data, especially when they work in critical environments.

Due to the reliability of the system and the coverage of laboratory procedures, the new EurOPDX Research Infrastructure adopted LAS as a LIMS for data management, with LAS to be installed across the six nodes of the distributed infrastructure (www.europdx.eu). Our lab is one of these nodes.

## Conclusion

The LAS platform is designed to assist researchers of biological and biomedical laboratories in all of their activities. The modular architecture manages heterogeneous and complex data and supports researchers and practitioners in performing different experimental procedures. The graphical interfaces and the web-based architecture are suitable for use in diverse environments, including hostile ones (e.g. in sterile conditions). Due to the modular architecture, a query module has been developed to perform federated queries over all the databases. Thanks to an intuitive graphical interface, the users can design complex queries without any knowledge of databases structures or query languages. In addition, a visualization tool has been integrated in the querying environment to generate targeted visual representations of biological entities and their relationships, allowing the scientists to extract relevant information for further analyses. More visualization tools will be envisioned in the near future.

## Notes

**Database URL:** https://github.com/lasircc/las-docker
